# Quinolone Allergy

**DOI:** 10.3390/pharmacy7030097

**Published:** 2019-07-19

**Authors:** Edoabasi U. McGee, Essie Samuel, Bernadett Boronea, Nakoasha Dillard, Madison N. Milby, Susan J. Lewis

**Affiliations:** 1 School of Pharmacy, Philadelphia College of Osteopathic Medicine, Suwanee, GA 30024, USA; 2 College of Pharmacy, University of Findlay, Findlay, OH 45840, USA; 3 Department of Pharmacy Practice, College of Pharmacy, University of Findlay, Findlay, OH 45840, USA; 4 Mercy Health—St. Anne Hospital, Toledo, OH 43623, USA

**Keywords:** quinolones, fluoroquinolones, allergy, hypersensitivity, cross-reactivity

## Abstract

Quinolones are the second most common antibiotic class associated with drug-induced allergic reactions, but data on quinolone allergy are scarce. This review article discusses the available evidence on quinolone allergy, including prevalence, risk factors, diagnosis, clinical manifestations, cross-reactivity, and management of allergic reactions. Although the incidence of quinolone allergy is still lower than beta-lactams, it has been increasingly reported in recent decades, most likely from its expanded use and the introduction of moxifloxacin. Thorough patient history remains essential in the evaluation of quinolone allergy. Many diagnostic tools have been investigated, but skin tests can yield false-positive results and in vitro tests have not been validated. The drug provocation test is considered the test of choice to confirm a quinolone allergy but is not without risk. Evidence regarding cross-reactivity among the quinolones is limited and conflicting. Quinolone allergy can be manifested either as an immediate or delayed reaction, but is not uniform across the class, with moxifloxacin posing the highest risk of anaphylaxis. Quinolone should be discontinued when an allergic reaction occurs and avoided in future scenarios, but desensitization may be warranted if no alternatives are available.

## 1. Introduction

Quinolones, highly effective synthetic antibiotics, are one of the most commonly prescribed antibiotic classes in outpatient [1] and in acute care hospital settings [2]. They inhibit DNA gyrase in Gram-negative bacteria and topoisomerase IV in Gram-positive bacteria, promoting the DNA cleavage and rapid killing of susceptible bacteria [3]. With their broad-spectrum activity against both Gram-positive and Gram-negative aerobic and anaerobic bacteria, excellent tissue and intracellular penetration, high bioavailability, and generally good oral tolerability, quinolones have a broad array of indications including treatment of urinary tract infections (UTIs), sexually transmitted diseases, gastrointestinal and abdominal infections, respiratory tract infections, bone and joint infections, and skin and soft tissue infections in adults. Although considered well-tolerated in general, quinolones can induce allergic reactions. In fact, they are the second most common antibiotics associated with allergic reactions, following beta-lactams [4,5,6]. Currently, most of the data regarding antibiotic allergy have been published for patients with a beta-lactam allergy and the paucity of data exist regarding quinolone allergy.

## 2. Structures

**Figure 1:**
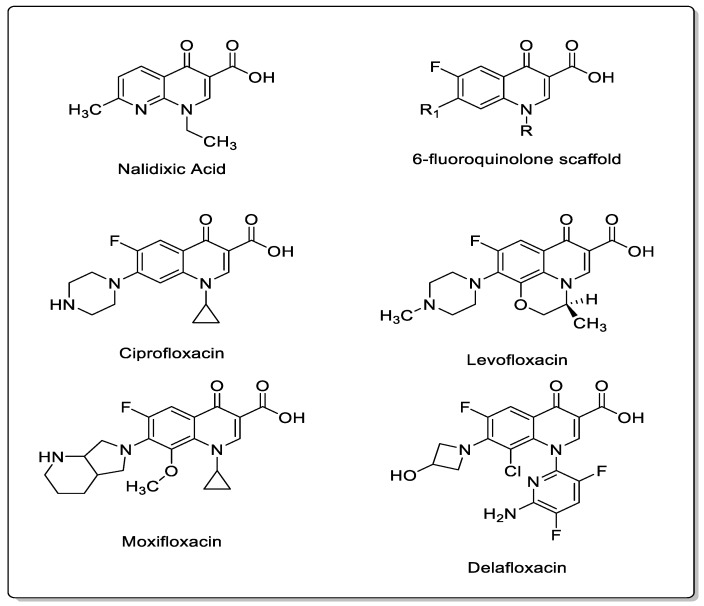
Chemical structures of commonly used quinolones.

Quinolones are classified into four generations based on chemical structure as well as antibacterial activity and pharmacokinetic properties [6]. The basic structure of quinolones consists of a bicyclic skeleton with the carboxylic acid and ketone groups at the third and fourth positions (Figure 1). The first quinolone agent, nalidixic acid, was approved by the Food and Drug Administration (FDA) to treat UTIs in 1964. In the 1980s, the fluorination of the original molecule yielded the fluoroquinolones, which substantially enhanced antibacterial spectrum and pharmacokinetic properties, sparking extensive studies and utilization of this class. Modification of substituents at positions N-1, C-7 and C-8 resulted in the production of various fluoroquinolones [7]. Currently, ciprofloxacin, levofloxacin, and moxifloxacin are the most commonly used quinolones in clinical practice while delafloxacin is the newest quinolone agent. Other quinolones are either used less frequently or have ceased to be used due to their toxicities [4]. Most quinolones are not subject to extensive hepatic metabolism except moxifloxacin, yet a small proportion of administered quinolones may be converted to reactive intermediates. It has been postulated that these partially metabolized intermediates may interact with proteins and generate covalent hapten-protein conjugates triggering immune reactions [8]. 

## 3. Prevalence

The true prevalence of quinolone allergy in the general population is unknown. Quinolone allergy with any severity attributed to 44 (95% CI: 34.8–53.3) emergency department visits per 100,000 prescriptions in the US between 2004 and 2010 based on the National Electronic Injury Surveillance System-Cooperative Adverse Drug Event Surveillance system and outpatient prescription data [9]. A recent retrospective study reported that quinolone allergy was reported in about 2% of hospitalized patients with most commonly documented reactions of hives, rash, and nausea/vomiting [10]. This incidence rate of quinolone allergy is lower than that of reported beta-lactam allergy (~10%) [11], but has been on the rise over the past decade [8,12]. This is most likely associated with the expanded use of quinolones and/or the introduction of moxifloxacin [4,8,13], which appears to be more immunogenic than other quinolones [9,14,15].

Quinolone allergy can be largely classified into two types: Ig-E mediated immediate reactions (IRs), which occur less than one hour after administration, and T-cell mediated delayed reactions (DRs), which occur more than an hour after administration. The most common quinolone allergies are IRs and ~70% of those cases are severe [8]. Anaphylaxis is an uncommon IR to quinolones and most cases were reported in the post-marketing stage. An estimated incidence of quinolone-induced anaphylaxis is 1.8–2.3 per 10,000,000 days of treatment [16]. Quinolones were reported to be responsible for 4.5% of 333 drug-induced anaphylaxis cases [17]. The risk of anaphylaxis may be different among quinolones. An in vitro laboratory evaluation and spontaneous adverse drug reaction reports in Europe has shown that moxifloxacin was most frequently involved in anaphylaxis (52.1–63%), followed by levofloxacin (13–35.7%) and ciprofloxacin (7.1–28.9%) [12,15,18]; however, a cohort study performed in the U.S. using a large insurance claim database found no difference in the incidence of anaphylaxis across the quinolone class [19], whereas the highest incidence of DRs has been observed with ciprofloxacin (33.3–34.9%), followed by levofloxacin (19.9–32.3%) and moxifloxacin (13.5–20.4%) [18,20].

## 4. Risk Factors

Information regarding common risk factors associated with quinolone allergies are limited. Blanca-Lopez and colleagues identified a previous history of beta-lactam allergy as a strong risk factor (OR:4.571; 95% CI: 0.987–21.171; adjusted OR: 23.654; 95% CI: 1.529–365.853) to develop a quinolone allergy [8]. Patients with a history of drug allergy were noted to be more susceptible to allergic reactions to a non-chemically related drug [21]. Another study displayed that 21% of patients with a history of IgE-mediated penicillin allergy reported allergic reactions to non-beta-lactams, compared to only 1% of patients with no drug allergy history [22]. 

Although the underlying mechanism is unknown, a genetic predisposition, or the fact that quinolones are likely to be selected as alternatives to treat those with a history of beta-lactam allergy, may explain these findings [23]. This is particularly of concern because beta-lactams and quinolones are often recommended as the first-line agents for many infections. Patients who are allergic to both classes are subject to receive inferior antibiotic therapy resulting in less favorable patient outcomes [6]. Additionally, a higher incidence of intravenous contrast allergy was noted in patients with a quinolone allergy (6%) compared to those with a penicillin allergy (2%) [10,24]. 

## 5. Evaluation and Diagnosis

The evaluation of drug allergy typically begins with a patient history and if a true allergy is suspected, appropriate tests are performed to confirm the diagnosis. However, the pathogenic mechanism of quinolone allergy is not clearly comprehended and no standardized or validated diagnostic tests have been determined, making its diagnosis challenging [25]. Thus, patient history remains an integral part of the quinolone allergy evaluation. Patient history should include the details of the symptoms, the timing of the reaction in relation to the causative agent, the timing since the reaction, and any relevant ingestions, taken concurrently and/or since the reaction [26]. Additionally, the patient’s current medication list, allergy history, and previous exposure to the same or any quinolone agent may be reviewed. A thorough patient history can aid to identify a true allergy, the type and severity of allergic reactions, and the need for diagnostic tests. Many patients who report quinolone allergy may not have a true allergy [26] but experience non-allergic adverse drug reactions such as gastrointestinal symptoms, central nervous system reactions, myalgias, and tendon rupture, which may not require further evaluation. The type of allergic reaction can be determined based on the time interval between the administration of the causative agent and the reaction [6].

In cases where patient history is not reliable, other diagnostic tests may be necessary to assess quinolone allergy. In recent years, multiple studies have been conducted to evaluate various diagnostic tests including skin tests, drug provocation tests (DPT), and in-vitro laboratory tests [24,27,28,29,30,31,32,33,34,35,36,37,38]. However, skin tests and in vitro tests have displayed low sensitivity and specificity, limiting their diagnostic utility [24,30,32,33,35,36,38].

### 5.1. Skin Tests

The value of skin testing remains a subject of debate. Both skin prick and intradermal skin tests are used for IRs. Skin prick tests are the safest and easiest but only moderately sensitive, while the intradermal skin tests are more sensitive but have a higher risk for inducing irritative and false-positive reactions. This can also lead to an anaphylactic reaction in IgE-dependent reactions [28]. Although some consider skin prick and intradermal tests useful, most studies show that quinolones can induce false-positive results, probably because of the capacity of some quinolones to directly induce histamine release because of mast cell activation [15,33]. It is also important to note that late or delayed reactions can occur with intradermal skin tests and should always be examined [28]. For prick and intradermal skin tests, widely divergent non-irritant test concentrations have been recommended, and desensitization may be possible in selected patients [31]. For patients with DRs, patch tests were performed on the upper back on unaffected, untreated, and uncleaned skin (no prior rinsing with alcohol) [28,33]. This patch was removed after one day and the test was read on days two, three, and four [28].

### 5.2. Drug Provocation Test

When all other diagnostic procedures are not available or lead to inconclusive results, a DPT is the only reliable tool to confirm or to rule out a quinolone allergy [27,29,33]. DPTs are considered the “gold standard” for establishing or excluding diagnosis of quinolone allergy, but they are time consuming and involve risks such as provocation of severe reactions [6]. Therefore, it is recommended that the procedure be performed after weighing the risks vs. the benefits and by trained personnel in a clinical setting where treatment for possible severe reactions is readily available [6,36]. Different doses and numbers of test doses have been utilized for a DPT, as depicted in Table 1 [5,6,8,15,25,32,33,34,36]. The initial dose is typically 1/100th of the usual full dose and is progressively increased to reach a therapeutic dose. If a test dose causes the original allergic reaction, a DPT result is considered positive, confirming quinolone allergy [29]. However, it should be noted that false-positive or false-negative results can still occur with a DPT. The absence of co-factors that contributed to the previous allergic reaction or the development of tolerance to the causative agent may lead to a false-negative result [6,29].

### 5.3. In Vitro Tests

Skin tests and/or DPTs are not always useful due to the potential risks for severe or life-threatening reactions and the high rate of false positive skin test results. In vitro tests offer a different, but valuable, approach to diagnose allergy to antibiotics. The most common in vitro tests that have been used with promising results for diagnosing immediate reactions to quinolones are radioimmunoassays (RIA) and the basophil activation test (BAT) [6,38]. These tests are recommended to be performed prior to in vivo tests in high-risk patients, including patients with a history of life-threatening reactions. RIA uses the high specificity of antibodies to target specific molecules and analyze their concentration in a sample [37], while BAT is a flow-cytometry-based functional assay that assesses the degree of cell activation after exposure to stimuli [38]. Currently, validated commercial in vitro tests for the evaluation of quinolone allergy are not available. Therefore, most studies have utilized tests that are produced in-house [6].

RIA has been shown to have low sensitivity for quinolones, varying from 31.6% to 54.5%, and high specificity [6]. This may be due to the different quinolones involved in each study and the severity of the reactions, as well as the total IgE levels, the time interval between the reaction and the performance of the test. Higher IgE levels were found in patients evaluated within a few months after the reaction, while patients showing negative results were generally evaluated after a longer time period [6]. These assays do not use enzymes and therefore reduce the risk of interference from the sample itself. The drawbacks of RIA include the use of a radiolabel and its short shelf life [37].

The utilization of BAT for drug allergy must be optimized for each drug because of the possible differences in the stimulation mechanism that leads to the upregulation of different activation markers [33]. Studies have shown the utility of BAT for evaluating quinolone-allergic reactions with sensitivity ranging from 50% to 100%, which can be explained by different factors [38]. Intrinsic factors such as patient medication therapy and the choice of activation markers can play a role in the results of BAT [33]. Sensitivity can be affected by both the quinolones involved in the reaction and the quinolones used for the test [38].

## 6. Cross-Reactivity of Quinolones

Cross-reactivity within the quinolone drug class has been reported in the literature, but the evidence is conflicting. A report by Dávila and colleagues was one of the initial reports describing cross-reactivity among quinolones with a recommendation to avoid any quinolone among patients who have had a reaction to one of them [39]. González and colleagues explored hypersensitivity among quinolones using skin tests and concluded that skin tests can predict group hypersensitivity, but not specific tolerance to each drug. This study found a high degree of cross-reactivity among fluoroquinolones, including moxifloxacin, which is chemically different from other quinolones [40]. Another case report described in vitro cross-reactivity between ciprofloxacin and ofloxacin, which could be due to the similarity in the structures of these drugs [41]. Anovadiya and colleagues described a cross-reactivity reaction between ciprofloxacin and levofloxacin in a seven-year-old male child with subacute appendicitis who was treated with ciprofloxacin and immediately developed multiple erythematous papules. An allergic reaction developed again when this patient was treated with levofloxacin. IgE binding at the seventh position of core structure of quinolones is likely to be the mechanism of a hypersensitivity reaction [42].

Conversely, a review of three case reports of IRs to moxifloxacin demonstrated a lack of cross-reactivity among moxifloxacin and ciprofloxacin. In the case reports, all three patients who developed IRs to moxifloxacin were able to tolerate the oral challenge tests to ciprofloxacin as well as a full course of ciprofloxacin. This lack of cross-reactivity may be explained by moxifloxacin’s unique side chain [43]. Another study reviewed 12 patients who had experienced an IR (four anaphylaxis and eight urticaria/angioedema) after oral administration of quinolones. Most of the ciprofloxacin-reactive patients tolerated levofloxacin and the majority of the levofloxacin-reactive patients tolerated ciprofloxacin. In addition, the patients who reacted to moxifloxacin tolerated ciprofloxacin and levofloxacin. This study highlighted the lack of cross-reactivity among quinolones and suggested that levofloxacin could be a safer alternative in cases of reaction to first-, second-, or fourth-generation quinolones [34].

Several studies have reviewed cross-reactivity within the quinolone drug class, but few have observed cross-reactivity among quinolones and other classes of antibiotics. A case was published to report a rare type of IR and a late phase reaction to an anti-tubercular therapy that consisted of ethambutol and levofloxacin. In this report, intradermal skin tests were performed; an immediate drug reaction was experienced with ethambutol within one hour, and a flare reaction occurred at the levofloxacin injection site 15 min after the test was administered and disappeared six hours later. The findings of this case suggest that drug eruptions are not necessarily caused by a single agent, and that multiple types of allergic reaction may occur consecutively in a single case [44].

## 7. Types and Manifestations of Quinolone Allergy

As previously mentioned, the mechanism of a quinolone allergy has been described mainly by two pathways based on the Coombs and Gell classification: IgE-mediated IR, also known as type I, and T-cell-mediated DR, also known as type IV responses [45]. The IgE-mediated pathological characteristic is mast-cell degranulation. Examples of IgE-mediated responses include urticaria, anaphylaxis, asthma, rhinitis and angioedema. In contrast, T-cell-mediated pathological characteristics may be induced by the subclassifications of type IV responses: Type IVa (monocyte activation), Type IVb (eosinophilic inflammation), Type IVc (CD4 or CD8-mediated killing of cells), Type IVd (neutrophil activation) and Type IVe (CD4 and CD8 activated T-cells). Examples of T-cell-mediated responses include contact eczema, maculopapular exanthema, bullous exanthema, Stevens–Johnson Syndrome Toxic epidermal necrolysis (SJS-TEN), fixed drug eruption (FDE), acute generalized exanthematous pustulosis (AGEP), and delayed urticaria. Other T-cell-mediated responses without a definitive Coombs and Gell classification include drug rash with eosinophilia and systemic symptoms (DRESS) and organ-specific T-cell-mediated responses like hepatitis and pneumonitis [45]. In addition to the two aforementioned pathways, a novel mechanism has recently been described of a non-IgE-mediated, pseudo-allergic anaphylactoid reaction of quinolone-induced anaphylaxis [46,47,48]. In this novel pathway, mast cells are activated via MAS-related G protein-coupled receptor-X2 (MRGPRX2), rather than Fcε receptors, which is linked to IgE-mediated IR [49]. Quinolones can activate the MRGPRX2, inducing non-IgE-mediated anaphylaxis, with a higher risk for patients with mastocytosis [46]. However, allergy manifestations due to quinolone use are not uniform across the class [24].

### 7.1. Moxifloxacin-Induced Allergy Manifestation

Among the quinolones, moxifloxacin has been reported to have the highest incidence of immediate allergic reactions, specifically anaphylaxis [4]. Published data of IgE mediated IR due to moxifloxacin have mainly consisted of case reports and short series. Urticaria was more frequently reported at a rate of 31.6–85% for patients on moxifloxacin as compared to anaphylaxis reported at a rate of 13–42% [4]. In a cross-sectional study of 54 patients in Istanbul, 17.5% of patients experienced an IR to moxifloxacin, of which 50% were anaphylaxis. In a Thailand retrospective review by Kulthanan and colleagues, a pattern of cutaneous reaction related to the administration of various quinolones was demonstrated. Three of 151 (2%) patients developed an allergic reaction to moxifloxacin; of those, one of three had an IR presenting with urticaria [50]. Interestingly, one female patient developed both an IR and delayed anaphylaxis due to moxifloxacin when orally provoked after a skin test [24].

T-cell-mediated DR induced by moxifloxacin was described in the case report where a patient with multiple drug allergies previously treated with levofloxacin and ciprofloxacin was prescribed moxifloxacin for a 10-day regimen. After four days of treatment, the patient developed a rash on the lower extremities, which progressed to oral thrush, skin sloughing, and shortness of breath [51]. In the aforementioned retrospective review by Kulthanan and colleagues where three of 151 (2%) patients developed a reaction to moxifloxacin, two developed DRs, with one developing SJS-TEN and the other a maculopapular rash [50].

### 7.2. Ciprofloxacin-Induced Allergy Manifestation

The literature suggests that ciprofloxacin is more commonly associated with DRs. Various reactions, including SJS-TEN, eczema, FDE, erythroderma, erythema multiforme and maculopapular rash were reported in 59 of 151 (39%) patients [50]. In addition, other cutaneous DR, such as demarcated erythematous plaques, vesicular and bullous lesions have also been reported in various case reports [52,53,54,55]. However, Kulthanan and colleagues described IRs due to ciprofloxacin in 20 of 151 (13%) patients, with urticaria being the most common, as observed in 12 of 20 (60%) patients [50].

Apart from the commonly noted IRs and DRs, Sim and colleagues describe a type II IgG-mediated allergic reaction which involved a patient who developed ciprofloxacin-induced thrombocytopenia. Immediately after two administered doses of intravenous ciprofloxacin, the patient’s platelet count decreased to 6000/μL from 220,000/μL and the platelet count continued to drop with five days of ciprofloxacin treatment. After the patient was switched to another antibiotic, the platelet count normalized (245,000/μL) during hospitalization and after discharge [56].

Giavina-Bianchi and colleagues recently reported a case on a woman with recurrent anaphylaxis who had five severe cases over a seven-year period. The first three episodes were induced by stinging insects while the other two were associated with ciprofloxacin. The reactions associated with ciprofloxacin included flushing, laryngeal edema and syncope. The patient was subsequently diagnosed seven years later with systemic mastocytosis, a disease associated with anomalous proliferation and accumulation of mast cells in different tissues, with a lifetime anaphylaxis risk of 50%. The authors concluded that the excess of mast cells and activation of MRGPRX2 receptors in patients with mastocytosis may induce anaphylaxis [46].

### 7.3. Levofloxacin-Induced Allergy Manifestation

The Thailand retrospective review noted that five of the 151 (3%) patients had an IR to levofloxacin with symptoms of urticaria, and nine of the 151 (6%) had a DR with a maculopapular rash and SJS/TEN [50]. Matsumoto and colleagues described a patient case in which the patient had both an IR and DR to levofloxacin [57]. Initially the patient received intravenous levofloxacin and skin pruritus occurred 30 min after initiation. Subsequently, levofloxacin 500 mg oral once daily was started and the skin pruritus resolved. However, three weeks later, the patient was hospitalized and found to have erythroderma, lymphadenopathy, pulmonary lesions and eosinophilia. Levofloxacin was discontinued, and supportive treatment was given, resulting in remission of all symptoms [57]. 

Nunez and colleagues reported a rather uncommon allergic reaction where a patient developed an IR to levofloxacin with generalized angioedema, urticaria and atypical symptoms of acute coronary syndrome evidenced by ST-segment decline [58]. Despite being previously healthy, the patient exhibited symptoms of severe heart failure and was diagnosed with type I Kounis syndrome, a case of a histamine-induced coronary artery spasm as a consequence of extensive vasodilation and low cardiac output [58]. 

### 7.4. Delafloxacin-Induced Allergy Manifestation

Delafloxacin, a newer generation quinolone, appears to cause similar allergic reactions in patients as other quinolones. In phase III clinical trials, seven of 741 patients treated with delafloxacin presented cutaneous tissue disorder such as pruritus, urticaria, dermatitis, and rash. No post-marketing data has been published regarding allergic reactions with delafloxacin [59]. 

## 8. Management

Quinolone allergy management is based on three foundational principles: discontinuation of the offending agent, initiation of alternative agent, and supportive care such as corticosteroid therapy, fluid replacement with electrolytes and albumin substitution. In addition, rescue agents such as corticosteroids, histamine antagonist, anti-IgE antibody, or short acting beta-adrenergic agonist can be utilized based on the clinical severity of the manifestation. A compromised airway would necessitate the use of an intravenous route as opposed to the oral route for treatment of the reaction. A series of case reports demonstrated successful management of quinolone allergies by the previously listed rescue agents. Moghaddam and colleagues described a DR to ciprofloxacin with progression to SJS where an oral prednisone taper starting at 60 mg daily was administered along with oral famotidine and diphenhydramine. Resolution was seen seven days after completion [60]. Other studies have documented resolution of allergic reaction due to ciprofloxacin using intramuscular epinephrine, intravenous corticosteroids, and oral chlorpheniramine [61,62]. Uzun and colleagues described the use of anti-IgE antibody in addition to standard rescue therapy to treat a patient with a past medical history (PMH) of Hepatitis C (HCV) who developed SJS-TEN after receiving one dose of levofloxacin [63]. The patient was treated with intravenous methylprednisolone 500 mg, human albumin, and intravenous omalizumab 300 mg, an anti-IgE monoclonal antibody [63]. TEN is typically related to an allergic drug reaction, however may occur occasionally after infections such as hepatitis. The authors hypothesized that having a PMH of HCV infection may have facilitated the immunologic reaction, causing an elevated eosinophil cationic peptide (ECP). They believed that ECP may have played an important role in the relationship between TEN and the immunologic reaction. Serum levels of IgE were monitored before and after omalizumab administration; dosing was based on pre-treatment serum levels of IgE. Symptoms were resolved after administration of omalizumab [63]. The novel mechanisms of drug-induced mast cell degranulation mediated by MRGPRX2 may modify the current management of drug hypersensitivity reactions potentially via personalized medicine. However, based on Giavina-Bianchi and colleagues’ report, patients with mastocytosis should avoid the use of quinolones due to their increased risk of hypesensitivity of up to 50% [46].

Utilizing these three foundational principles may work as treatment for most patients. However, in some cases with multiple confirmed antibiotic allergies, quinolones may be the only therapeutic option available, making de-sensitization a necessity. Desensitization has been performed for both IRs and DRs, but mainly IRs due to ciprofloxacin [64]. To avoid lapses in drug administration and the development of a systemic reaction, it is recommended to administer the quinolone in progressive doses at intervals of 30–60 minutes until the therapeutic dose is achieved [64,65,66,67]. Lastly, serious delayed cutaneous reactions may require wound care management or surgical debridement based on the extent of the severity [45].

## 9. Summary and Recommendations

Quinolones are the second most common antibiotic class associated with allergic reactions. The true prevalence of quinolone allergies still remains unknown, but its incidence has increased in recent decades, likely due to the extensive utilization and the introduction of moxifloxacin. The literature suggests that individuals with a history of allergy to beta-lactams allergy or intravenous contrast are more prone to developing a quinolone allergy. Diagnosis of a quinolone allergy is difficult due to its unknown pathogenic mechanism and lack of validated diagnostic tests. Skin prick tests can yield false positive results and in vitro tests such as RIA and BAT have not been validated for routine clinical use. DPTs are the diagnostic tests of choice for establishing or excluding a diagnosis of quinolone allergy but are time consuming and also involve risks. A thorough patient history is therefore essential to assess quinolone allergy. There is conflicting evidence regarding cross-reactivity among the quinolone drug class. Several studies have established cross-reactivity among different quinolones, while tolerance to a different quinolone in patients with a reported allergy have been published as well. Allergic manifestations due to quinolone use is not uniform across the class, though moxifloxacin has been implicated in causing anaphylaxis more frequently than others. Overall, IR has frequently been reported as most common in the previous literature, but many case reports and case series describe DR induced by quinolones. In terms of management of quinolone allergy, the mainstay is to ensure discontinuation of the offending agent and appropriate documentation in patient medical record. Initiating an alternative agent and providing supportive care may also be warranted. Patients with a known history of mastocytosis may avoid quinolones. Desensitization may be required in patients with no other antibiotic options. 

## Figures and Tables

**Table 1:** Skin test and drug provocation testing concentrations for commonly used quinolones [5,6,8,15,25,32,33,34,36].

Drug	Skin Prick Test (mg/mL)	Intradermal Test (mg/mL)	Drug Provocation Test (mg)
Ciprofloxacin	0.02–5.0	0.005–0.05	5-50-100-150-200 (5 doses) 50-125-250-500 (4 doses)
Levofloxacin	0.025–5.0	0.005–0.05	5-50-100-150-200 (5 doses) 50-125-250-500 (4 doses)
Moxifloxacin	1.0–20.0 or 400 mg tablet suspended in saline solution (more common)	0.004–0.05	5-50-100-100-150 (5 doses) 25-50-100-200 (4 doses)
